# Effects of the Surface Charge Density of Clay Minerals on Surface-Fixation Induced Emission of Acridinium Derivatives

**DOI:** 10.1021/acsomega.1c03157

**Published:** 2021-08-13

**Authors:** Yuma Yoshida, Tetsuya Shimada, Tamao Ishida, Shinsuke Takagi

**Affiliations:** † Department of Applied Chemistry for Environment, Graduate School of Urban Environmental Sciences, Tokyo Metropolitan University, 1-1 Minami-ohsawa, Hachioji-shi, Tokyo 192-0397, Japan; ‡ Research Center for Gold Chemistry, Tokyo Metropolitan University, 1-1 Minami-ohsawa, Hachiohji-shi, Tokyo 192-0397, Japan; § Research Center for Hydrogen Energy-based Society (ReHES), Tokyo Metropolitan University, 1-1 Minami-ohsawa, Hachiohji-shi, Tokyo 192-0397, Japan

## Abstract

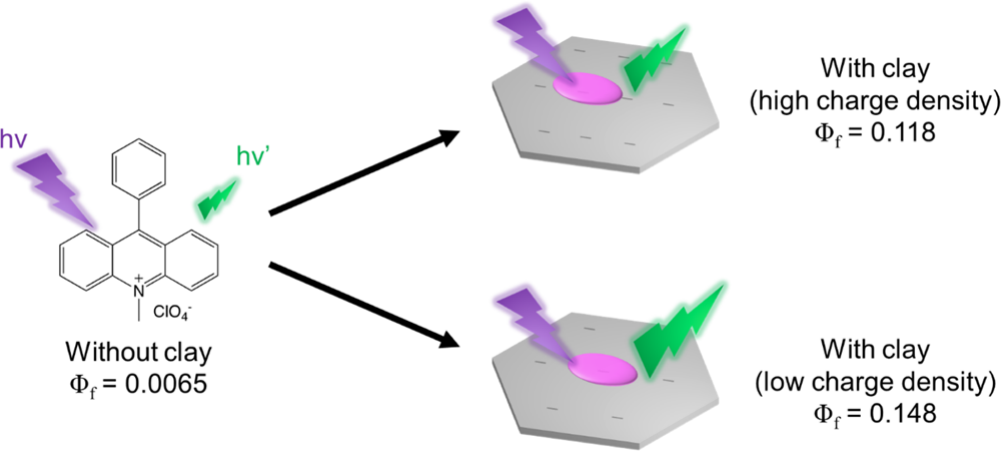

Surface-fixation induced emission is a fluorescence enhancement phenomenon, which is expressed when dye molecules satisfy a specific adsorption condition on the anionic clay surface. The photophysical behaviors of two types of cationic acridinium derivatives [10-methylacridinium perchlorate (Acr^+^) and 10-methyl-9-phenylacridinium perchlorate (PhAcr^+^)] on the synthetic saponites with different anionic charge densities were investigated. Under the suitable conditions, the fluorescence quantum yield (Φ_f_) of PhAcr^+^ was enhanced 22.3 times by the complex formation with saponite compared to that in water without saponite. As the inter-negative charge distance of saponite increased from 1.04 to 1.54 nm, the Φ_f_ of PhAcr^+^ increased 1.25 times. In addition, the increase in the negative charge distance caused the increase in the integral value of the extinction coefficient and the radiative deactivation rate constant (*k*_f_) and the decrease in the nonradiative deactivation rate constant. It should be noted that the 2.3 times increase in *k*_f_ is the highest among the reported values for the effect of clay. From these results, it was concluded that the photophysical properties of dyes can be modulated by changing the charge density of clay minerals.

## Introduction

Organic fluorescent dyes have attracted great attention as probes, sensors, display materials such as organic light-emitting diodes (OLEDs) and fluorescent sheets, and even as optoelectronics materials.^1−7^ Organic dyes have been widely used in these fields because they are composed of universal elements such as C, N, and O and have less environmental impact compared with inorganic phosphors containing transition elements,^8,9^ and typical quantum dots contain harmful substances such as Cd and In.^10−12^ In devices such as OLEDs and fluorescent sheets, organic dyes are expected to be used in a solid state. On the other hand, the problem is that the incorporation of dyes with a highly flat π-conjugated system into solids provoke self-fluorescence quenching due to an aggregation by π–π interaction.^13,14^ Some aggregates, such as cyanine dyes, show a high fluorescence quantum yield due to the formation of J-aggregates.^15,16^ In addition, AIE (aggregation-induced emission)-active dyes whose fluorescence quantum yield is enhanced by suppressing the intramolecular vibration and rotation by forming aggregates have been reported.^17,18^ However, accurate molecular design and synthesis techniques are required for these AIE-active dyes.

We have found a phenomenon called surface-fixation induced emission (S-FIE) in which the fluorescence of the dye is enhanced by a complex formation with nanosheets such as clay minerals.^19−22^ Clay minerals are typical layered materials and have a two- or three-sheet structure in which tetrahedral sheets and octahedral sheets are stacked. The isomorphic substitution of Si^4+^ by Al^3+^ in the tetrahedral sheet or Al^3+^ by Mg^2+^ in the octahedral sheet produces negative charges on the surface of clay minerals. The structure of typical clay minerals is shown in Figure S1. Clay minerals have been used for various purposes because they have properties such as ion adsorption capacity, swelling property, and thermal stability, and these are naturally ubiquitous materials.^23−25^ Furthermore, clay minerals as host materials have received much attention in recent years. Since clay minerals have a flat surface at the atomic level, can be exfoliated into a single layer, and have optical transparency in a solution state, they have been investigated as photo-functional host materials.^26−35^ Although fluorescence enhancement by complexing clay minerals and methyl viologen was reported in 1986,^36,37^ the systematic study was not conducted since an aggregation formation of dyes on the clay surface easily takes place.^38−40^ However, we found a phenomenon called the size-matching effect, in which the specific cationic molecules such as multi-cationic porphyrins are adsorbed on the clay surface without aggregation.^30,41^ This report enabled and inspired the researches to focus on the intrinsic photochemical behavior of various dyes on the clay minerals without aggregation.

**Figure 1:**
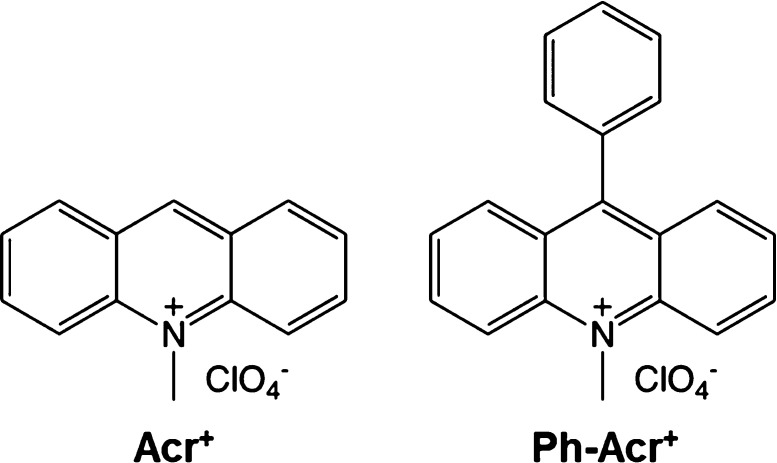
Structure of acridinium derivatives (Acr^+^ and PhAcr^+^).

S-FIE is the fluorescence enhancement phenomenon due to the adsorption of dyes on the flat clay surface.^19,22,42^ In this phenomenon, (i) a decrease in the nonradiative deactivation rate constant due to the suppression of molecular motion such as intramolecular vibration and rotation and/or (ii) the increase in the radiative deactivation rate constant due to the resembling molecular structures between the ground and excited states are the causes of fluorescence enhancement. S-FIE has a similar aspect to AIE. S-FIE has the advantage that various cationic dyes can be applied with certain expectation. In addition, the host material, clay mineral, is a naturally ubiquitous material. Several researches have reported fluorescence enhancement due to the complexation of various dyes and clay minerals.^19−22,35,42^ Although these reports have investigated the effect of the dye structure on the enhancement of the fluorescence quantum yield, little has been reported on the effect of clay minerals as host materials. This paper reports and discusses the photophysical behavior and fluorescence enhancement of mono-cationic acridinium derivatives (Acr) on the clay surface by using two acridinium derivatives as guest molecules (Figure 1) and four synthetic saponites which have different negative charge densities as host materials.

## Experimental Section

### Materials

Clay minerals (synthetic saponites): Sumecton SA (Sap1.2) was purchased from Kunimine Industries Co., Ltd. and was used without further purification. The synthetic saponites, named Sap1.0, Sap1.4, and Sap1.6, were synthesized by hydrothermal synthesis according to a previous paper.^41^ The synthetic saponites were analyzed with atomic force microscopy, X-ray diffraction, X-ray fluorescence, and Fourier transform infrared spectroscopy, as described in the previous paper.^41^ The general structure and chemical formulas of synthetic saponites are shown in Figure S1 and Table S1. According to the paper, the cation-exchange capacity (CEC) values of Sap1.2, Sap1.0, Sap1.4, and Sap1.6 were 0.99, 1.32, 0.69, and 0.59 mequiv g^–1^, respectively. Since the theoretical specific surface area of the synthetic saponite is 750 nm^2^ g^–1^, the negative charge distances of Sap1.2, Sap1.0, Sap1.4, and Sap1.6 were calculated to be 1.20, 1.04, 1.45, and 1.57 nm on the basis of a hexagonal array, respectively. The aqueous dispersion of saponite nanosheets, whose particle size is small (<100 nm), is substantially transparent in the UV–visible range. Water was deionized with an ORGANO BB-5A system (PF filter ×2 + G-10 column). 10-Methyl-9-phenylacridinium perchlorate was purchased from Tokyo Kasei. 10-Methylacridinium methyl sulfate was purchased from Aldrich. The counter ion was changed to perchlorate with an ion-exchange resin (Organo, Amberlite resin IRA-400 treated with HClO_4_).

### Analysis

Thermogravimetry–differential thermal analysis curves were measured with a Shimadzu DTG-60H analyzer to determine the water content of Acr and synthetic saponites. The temperature was ramped from room temperature to 120 °C with a heating rate of 10 °C/min under dry air as a purge gas and was held for 60 min. Absorption spectra were obtained on a UV-3150 UV–vis spectrophotometer (SHIMADZU). Fluorescence spectra were obtained on an FP-6500 spectrofluorometer (Jasco), and the excitation light was set at the absorption maximum wavelength of each sample. The reproducibility and signal-to-noise ratio of the fluorescence intensity are 0.5% and 100:1 or higher, respectively. The fluorescence quantum yield was determined by the relative method. Rhodamine 6G was used as a standard for the calculation of the fluorescence quantum yield of Acr with and without synthetic saponites. The fluorescence quantum yield of Rhodamine 6G in water is 0.90.^43^ Fluorescence lifetime was measured by a C4780 picosecond fluorescence lifetime measurement system (Hamamatsu Photonics). A Nd^3+^ YAG laser (EKSPLA PL2210JE + PG-432, fwhm 25 ps, 1 kHz) was used for excitation. The excitation wavelength was 355 nm. The fluorescence lifetimes were calculated by deconvoluting the excitation pulse in each measurement range.

### Sample Preparation

Acr stock solutions were prepared in a concentration range of 1.0 × 10^–3^ to 1.0 × 10^–4^ M. Stock dispersions of synthetic saponites were prepared in a concentration range of 1.0 × 10^–3^ to 1.0 × 10^–4^ equiv L^–1^. To prepare Acr–clay complexes, the above aqueous stock solutions were mixed at an arbitrary rate and were diluted with water under stirring in a quartz cell (1.0 × 1.0 cm). UV–vis absorption spectra were measured under the concentration of 1 × 10^–6^ M for Acr and 1 × 10^–3^ equiv L^–1^ for synthetic saponites. Fluorescence spectra were measured under the concentration of 3.33 × 10^–9^ M for Acr and from 2.0 × 10^–4^ to 3.33 × 10^–7^ equiv L^–1^ for synthetic saponites. Fluorescence lifetime were measured under the concentration of 4.0 × 10^–8^ M for Acr and 4.0 × 10^–4^ equiv L^–1^ for synthetic saponites.

## Results and Discussion

### Absorption Behavior of Acr in Water and on the Clay Surface

**Figure 2:**
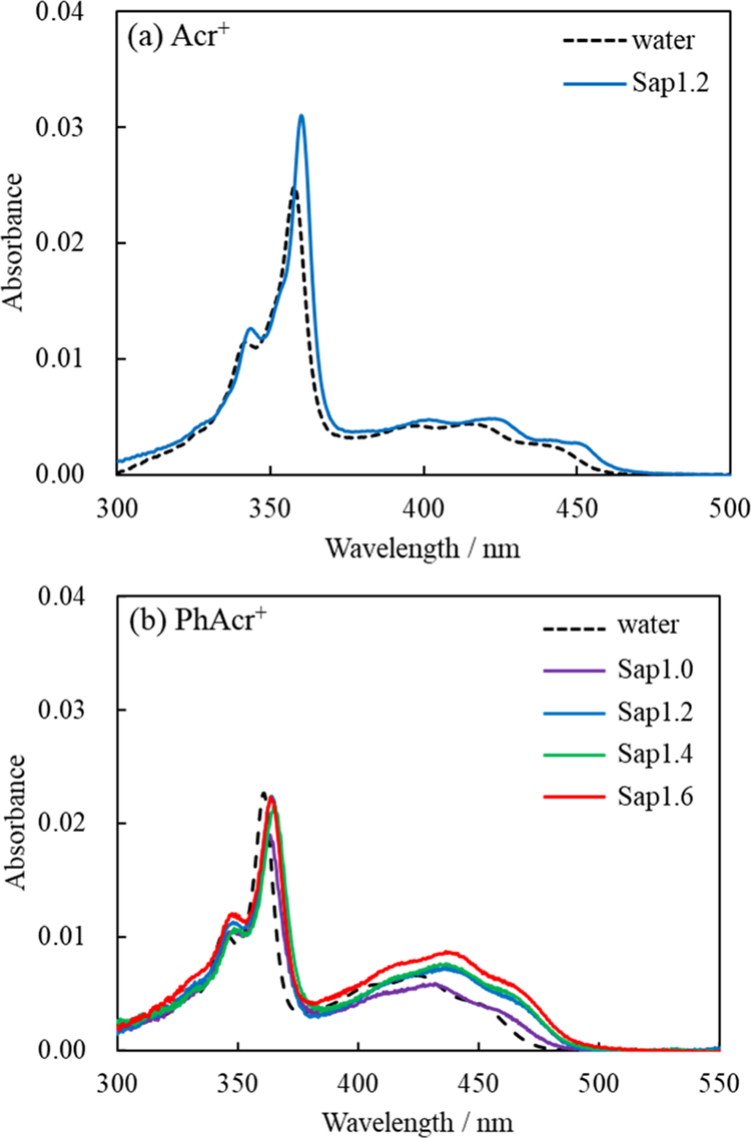
UV–vis absorption spectra of Acr (a) Acr^+^ and (b) PhAcr^+^ with and without clay in aqueous solution. [Acr] = 1.0 × 10^–6^ M and [Sap] = 1.0 × 10^–3^ equiv L^–1^.

The adsorption behavior of Acr on various synthetic saponites was evaluated by measuring UV–vis absorption spectra. The UV–vis absorption spectra of Acr^+^ and PhAcr^+^ with and without clay in water are shown in Figure 2. The band maxima of absorption (λ_ab_) are summarized in Table 1.

**Table 1:** Band Maxima of Absorption(λ_ab_) and Fluorescence(λ_fl_) and Stokes Shift (Δλ)^a^ of Acr with and without Clay in Water

compound	environment	λ_ab_/nm (cm^–1^)	λ_fl_/nm (cm^–1^)	Δλ/cm^–1^
Acr^+^	water	444 (22,523)	462 (21,645)	878
Acr^+^	Sap1.2	448 (22,321)	465 (21,505)	816
PhAcr^+^	water	454 (22,026)	485 (20,619)	1408
PhAcr^+^	Sap1.0	460 (21,739)	490 (20,408)	1331
PhAcr^+^	Sap1.2	465 (21,505)	490 (20,408)	1097
PhAcr^+^	Sap1.4	465 (21,505)	490 (20,408)	1097
PhAcr^+^	Sap1.6	468 (21,368)	490 (20,408)	959

a Stokes shift is defined as λ_ab_ – λ_fl_.

Both Acr^+^ and PhAcr^+^ showed a red shift by the adsorption on the clay surface. The wavelength shift width of Acr^+^ on Sap1.2 was 4 nm (from 444 to 448 nm) while that of PhAcr^+^ on Sap1.2 was 11 nm (from 454 to 465 nm). Such spectral shifts on the clay surface have been reported in many papers.^28−30,36,46,47^ It was proposed that the cause of the red shift is that the π-conjugated system is expanded by a flattening of molecules on the clay surface, which is flat at the atomic level.^44−47^ Since PhAcr^+^, which has a rotational substituent, showed a larger wavelength shift than Acr^+^, which does not have a rotational substituent, it is presumed that a similar phenomenon occurred in the case of Acr. Furthermore, as the negative charge distance of the clay increased, the red shift of the absorption maxima of PhAcr^+^ increased. It is considered that the increase in the adsorption strength due to the increase in the inter-negative charge distance on the clay surface induced the further red shift.

Acridinium derivatives have two main bands in their absorption spectra, as shown in Figure 1. It is known that a very narrow absorption band at about 360 nm is attributed to the S_0_–S_2_ transition and a broad absorption band at about 450 nm is attributed to the S_0_–S_1_ transition.^48,49^ To discuss the S_0_–S_1_ transition, the integral values of the extinction coefficient at 380–550 nm are summarized in Table 2. The integral values of the extinction coefficient of Acr on the clay surface were larger than those in water (Table 2) in most cases, indicating an increase in the transition probability, namely, the Franck–Condon factor, when Acr is adsorbed on the clay surface. This result suggests that the difference of nuclear coordinates of the ground and excited states become smaller by the adsorption of Acr on the clay surface. As the inter-negative charge distance on the clay surface increased, the integral value of the extinction coefficient (ε) of PhAcr^+^ increased. Since the clay surface with a larger inter-negative charge distance becomes more hydrophobic, it is presumed that the Acr adsorbed on a more hydrophobic surface was more firmly fixed. Similar to these, the photochemical properties of PhAcr^+^ such as λ_ab_ and ε were affected by the adsorption on the clay surface, and the effect of the clay surface was larger as the inter-negative charge distance on the clay surface increased. These indicate that the hydrophobic interaction between PhAcr^+^ and the clay surface plays an important role in the adsorption and photochemical properties of PhAcr^+^ on the clay surface.

**Table 2:** Integral Values of Extinction Coefficients of Acr with and without Various Clays in Water^a^

		integral of the extinction coefficient/10^7^ M^–1^ cm^–1^		
compound	clay minerals	∫ε^W^	∫ε^C^	∫ε^C^/∫ε^W^
Acr^+^	Sap1.2	1.46	1.74	1.19
PhAcr^+^	Sap1.0	2.39	2.25	0.94
PhAcr^+^	Sap1.2	2.39	2.82	1.18
PhAcr^+^	Sap1.4	2.39	2.99	1.25
PhAcr^+^	Sap1.6	2.39	3.26	1.37

a The integral range is 18,182–26,315 cm^–1^ (380–550 nm). ∫ε^W^ and ∫ε^C^ are the integral values of the extinction coefficients of Acr in water and with clays, respectively.

### Fluorescence Behavior of Acr in Water and on the Clay Surface

**Figure 3:**
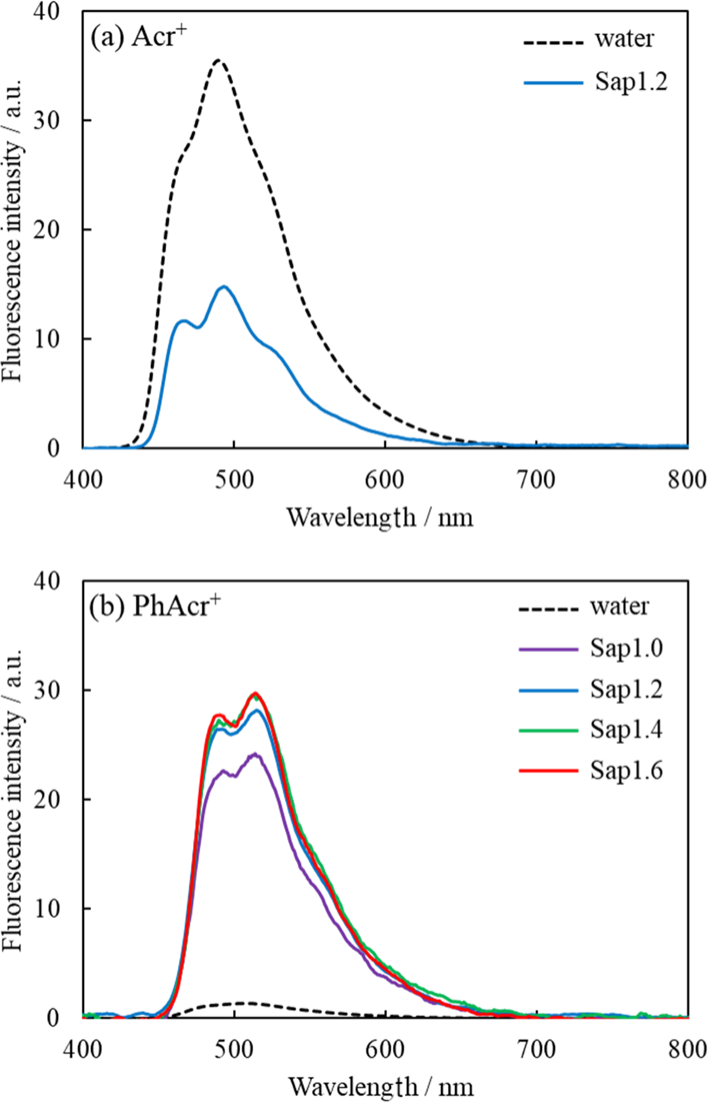
Fluorescence spectra of Acr [(a) Acr^+^ and (b) PhAcr^+^] with and without clays in water. [Acr] = 3.33 × 10^–9^ M and [Sap] = 3.33 × 10^–5^ equiv L^–1^. The excitation wavelength was 360 nm. The spectra were corrected with the absorbance at 360 nm.

The fluorescence behavior of Acr on various synthetic saponites was evaluated by measuring the fluorescence spectra. The fluorescence spectra of Acr^+^ and PhAcr^+^ with various clays in water at each loading level are shown in Figure S2. The fluorescence intensity at each loading level is shown in Figure S3. As most dyes suffered self-fluorescence quenching when these are adsorbed on the clay surface,^50−53^ Acr was self-quenched when it was adsorbed on the clay surface as well. Meanwhile, Acr was not self-quenched when the loading level of Acr is less than 0.01% vs CEC in any combination of Acr and clays. The fluorescence spectra of Acr in water and on the clay surface (0.01% vs CEC) are shown in Figure 3. The fluorescence maxima (λ_fl_) and Stokes shifts (Δλ) are summarized in Table 1.

In most cases, organic molecules do not show a clear vibrational structure in the fluorescence spectra in solution because of (i) the modulation of the molecular structure and the surrounding solvent orientation and (ii) the difference of the molecular structure and solvent reorientation in the ground and excited states. Actually, the fluorescence spectra of Acr^+^ and PhAcr^+^ in water did not show clear vibrational structures, as can be seen in Figure 3 (broken line). On the other hand, both Acr^+^ and PhAcr^+^ showed a clearer vibrational structure in the fluorescence spectra due to the adsorption on the clay surface. For all cases, the Stokes shift of Acr on the clay surface became smaller than that in water, as can be seen in Table 1. The Stokes shift indicates the degree of solvent reorientation around molecules at the electronic transition.^54^ It is known that hydrophobic interaction has an important role in the adsorption of organic dyes on the clay surface.^55,56^ Thus, when a dye molecule is adsorbed on the clay surface, the number of surrounding water molecules becomes almost half compared to that in water because half of the Acr surface is covered by the clay surface. It suggests that the dyes on the clay surface have less solvent reorientation in the electronic transition, in addition to less molecular structure changes in the electronic transition. Similar to these, the reasons why a clear vibrational structure was observed and the Stokes shift was small when Acr was adsorbed on the clay surface are the fixation of the molecular structure and less solvent reorientation in the electronic transition. In addition, as the negative charge distance on the clay surface increased, the Stokes shift tended to decrease. The increase in the negative charge distance on the clay surface makes the clay surface more hydrophobic.^57,58^ Acr should be adsorbed more parallelly on the clay surface for a more hydrophobic clay surface because of the effective hydrophobic interaction between Acr and the clay surface. A strong fixation of Acr on the clay surface causes the decrease in the molecular structure relaxation in the excited state. Therefore, the decrease in the Stokes shift with the increase in the inter-negative charge distance is attributed to a decrease in the structural change and the solvent relaxation of Acr in the excited state.

Fluorescence quantum yields (Φ_f_) of Acr^+^ and PhAcr^+^ in water and on various clays are shown in Table 3. Although Φ_f_ of Acr^+^ on the clay surface became smaller than that in water, Φ_f_ of PhAcr^+^ was enhanced approximately 20 times by the adsorption on the clay surface. As the negative charge distance on the clay surface increased, Φ_f_ of PhAcr^+^ increased. It is well-known that Φ_f_ of dyes on the clay surface is much enhanced compared to that in solution when the dye is adsorbed without aggregation. The effect of the clay surface on the fluorescence enhancement is called S-FIE.^29,42^ Several studies have reported that the major factor for the fluorescence enhancement is a fixation of rotational substituents.^21,22^ Thus, a strong fixation of Acr on the hydrophobic clay surface, suppressing the rotation of the substituent, could be a reason why the hydrophobic clay induced the large fluorescence enhancement, as in the case with the discussion for the Stokes shift.

**Table 3:** Fluorescence Quantum Yield (Φ_f_) and Fluorescence Lifetime (τ) of Acr with and without Clays in Water^a^

		quantum yield			fluorescence lifetime/ns		
compound	clay minerals	Φ_f_^W^	Φ_f_^C^	Φ_f_^C^/Φ_f_^W^	τ^W^	τ^C^	τ^C^/τ^W^
Acr^+^	Sap1.2	0.173	0.072	0.42	30.2	28.2	0.93
PhAcr^+^	Sap1.0	0.0065	0.118	18.1	1.55	14.5	9.4
PhAcr^+^	Sap1.2	0.0065	0.137	21.1	1.55	15.8	10.2
PhAcr^+^	Sap1.4	0.0065	0.144	22.2	1.55	15.1	9.7
PhAcr^+^	Sap1.6	0.0065	0.148	22.3	1.55	16.1	10.4

a Φ_f_^W^ and Φ_f_^C^ are the Φ_f_ values of Acr in water and on the clay surface, respectively. τ^W^ and τ^C^ are the τ values of Acr in water and on the clay surface, respectively.

To further discuss the photophysical behavior of Acr on the clay surface, a time-resolved fluorescence measurement was carried out. Fluorescence lifetimes of Acr^+^ and PhAcr^+^ in water and on various clays are shown in Table 3. Fluorescence decays are shown in Figure S4. The radiative deactivation rate constants (*k*_f_) and the nonradiative deactivation constants (*k*_nr_) are calculated from the fluorescence lifetime (τ) and fluorescence quantum yield (Φ_f_) according to eqs 1 and 2 and are shown in Table 4.
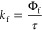
1
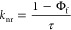
2

**Table 4:** Radiative (*k*_f_) and Nonradiative (*k*_nr_) Deactivation Rate Constants of Acr with and without Various Clays in Water^a^

		radiative deactivation rate constant/10^7^ s^–1^			nonradiative deactivation rate constant/10^7^ s^–1^		
compound	clay minerals	*k*_f_^W^	*k*_f_^C^	*k*_f_^C^/*k*_f_^W^	*k*_nr_^W^	*k*_nr_^C^	*k*_nr_^C^/*k*_nr_^W^
Acr^+^	Sap1.2	0.57	0.26	0.45	2.7	3.3	1.2
PhAcr^+^	Sap1.0	0.42	0.81	1.9	64.1	6.1	0.095
PhAcr^+^	Sap1.2	0.42	0.87	2.1	64.1	5.5	0.085
PhAcr^+^	Sap1.4	0.42	0.95	2.3	64.1	5.7	0.088
PhAcr^+^	Sap1.6	0.42	0.90	2.2	64.1	5.3	0.083

a *k*_f_^W^ and *k*_f_^C^ are the *k*_f_ values of Acr in water and on the clay surface, respectively. *k*_nr_^W^ and *k*_nr_^C^ are the *k*_nr_ values of Acr in water and on the clay surface, respectively.

**Figure 4:**
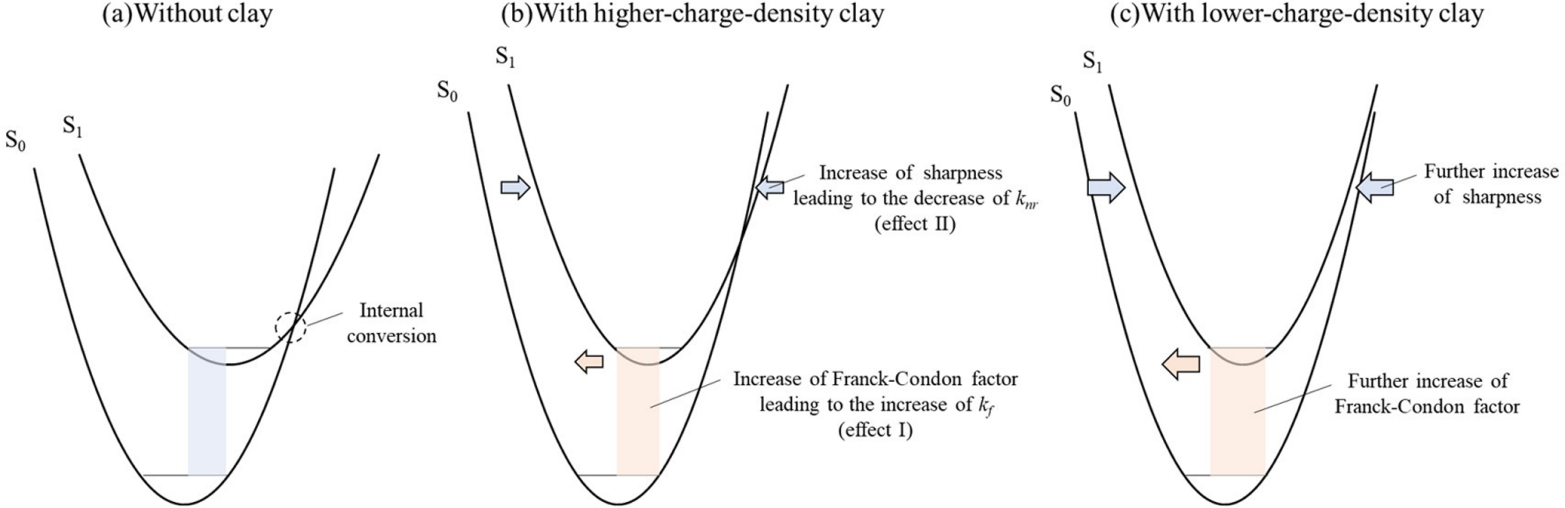
Plausible conceptual potential energy curves of the ground and excited states for PhAcr (a) without clay, (b) with higher-charge-density clay, and (c) with lower-charge-density clay.

When Acr^+^, which has no rotational substituents, was adsorbed on the clay surface, *k*_f_ decreased. In other words, *k*_f_^C^ was smaller than *k*_f_^W^ in the case of Acr^+^. On the other hand, *k*_f_ increased around 2-fold and *k*_nr_ decreased by about one-tenth when PhAcr^+^, which has a rotational substituent, was adsorbed on the clay surface. It is known that *k*_f_ and *k*_nr_ tend to increase and decrease by the adsorption on the clay surface, respectively, and these lead to the fluorescence enhancement (S-FIE).^22^ A previous report indicated that the change in the potential energy curve for the ground and excited states of adsorbed species upon the adsorption on the clay surface causes such changes in *k*_f_ and *k*_nr_.^22^ According to the paper, it is expected for the dyes on the clay surface that (i) the most stable structure is relatively similar between the ground and excited states (effect I) and (ii) the potential energy curve is relatively sensitive against the nuclear coordinates (effect II), compared to those without clay, as can be seen in Figure 4a,b. It was concluded that effect I and II tend to increase *k*_f_ and decrease *k*_nr_. As mentioned above, PhAcr^+^ has a rotational substituent at the 9-position, while Acr^+^ has no rotational substituents. Thus, PhAcr^+^ is more sensitive than Acr^+^ against a surrounding environmental change. Consequently, the intramolecular rotation of PhAcr^+^ was suppressed by the clay surface, and then, *k*_f_ increased when PhAcr^+^ was adsorbed on the clay surface (effect I). The increase in the integral values of the extinction coefficient by the adsorption on the clay surface (Table 2) can be rationalized in the same way. In the case of *k*_nr_, the decrease in *k*_nr_ on the clay surface is attributed to the suppression of the mobility of the rotational substituent due to the adsorption on the clay surface (effect II). It should be noted that the 2.3 times increase in *k*_f_ induced by effect I is the highest among the reported values.

As the negative charge distance on the clay surface increased, *k*_f_ and *k*_nr_ tended to increase and decrease upon the adsorption on the clay surface, respectively. These results indicate that effect I and II were enhanced by the increase in the negative charge distance on the clay surface, as shown in Figure 4c. As mentioned above, the increase in the inter-negative charge distance on the clay surface caused the increase in the extinction coefficient and the red shift of λ_max_ for PhAcr^+^ on the clay surface (Table 2). These results consistently indicate that the interaction between PhAcr^+^ and the clay surface becomes stronger as the inter-negative charge distance on the clay surface is increased. Since clays with a large inter-negative charge distance have a highly hydrophobic surface, the hydrophobic interaction causes PhAcr^+^ to be more strongly immobilized on the clay surface. In conclusion, the present study has demonstrated that the photophysical behavior of Acr on clays was affected by not only the guest molecular structure but also the structure of clay minerals, which is the host material.

## Conclusions

In this paper, photophysical behaviors of cationic Acr with and without various clays were investigated. The absorption maxima showed a red shift when Acr was adsorbed on the clay surface. The expansion of the π-conjugated system was suggested because the absorption spectra of PhAcr^+^, which has a rotational substituent, showed a longer red shift than that of Acr^+^. In addition, an increase in the extinction coefficient, the vibrational structure in the fluorescence spectra, and the enhancement of Φ_f_ were observed for PhAcr^+^ when PhAcr^+^ was adsorbed on the clay surface. Furthermore, an increase in *k*_f_ and a decrease in *k*_nr_ were observed. These changes of photophysical properties indicate the suppression of the mobility of the rotational substituent by the flattening of the dihedral angle between the rotational substituent and acridinium ring when PhAcr^+^ was adsorbed on the clay surface. A decrease in the Stokes shift of PhAcr^+^ on the clay surface indicates that the effect of solvent relaxation was decreased due to the decrease in the molecular structure and the solvation relaxation at the excited state when PhAcr^+^ was adsorbed on the clay surface. The present results suggest that not only the guest dye structure but also the clay structure as host materials affected the photophysical property of the dyes adsorbed on the clay surface.

## Supporting Information Available

General structure and chemical formulas of synthetic saponites, fluorescence spectra of Acr^+^ and PhAcr^+^ with various clays in water at each loading level, fluorescence intensity at each loading level, and fluorescence decay profiles for Acr^+^ and PhAcr^+^ with various clays in water (PDF)
