# Supplementary material for: Effects of the Surface Charge Density of Clay Minerals on Surface-Fixation Induced Emission of Acridinium Derivatives
Source: ACS Omega. 2021 Aug 13;6(33):21702–8. doi: 10.1021/acsomega.1c03157 (PMC8388081; doi:10.1021/acsomega.1c03157)
Supplement: Supplementary file 1 — ao1c03157_si_001.pdf [file ao1c03157_si_001.pdf]

[Supporting information]

Effects of the Surface Charge Density of Clay Minerals on Surface-Fixation Induced Emission of Acridinium Derivatives

*Yuma Yoshida,[1] Tetsuya Shimada,[1,3] Tamao Ishida,[1,2,3] Shinsuke Takagi[1,3] **

[1]Department of Applied Chemistry for Environment, Graduate Course of Urban Environmental Sciences, Tokyo Metropolitan University, Minami-ohsawa 1-1, Hachiohji, Tokyo 192-0397 Japan

[2] Research Center for Gold Chemistry, Tokyo Metropolitan University, 1-1 Minami-ohsawa, Hachiohji-shi, Tokyo 192-0397, Japan

[3] Research Center for Hydrogen Energy-based Society (ReHES), Tokyo Metropolitan University, 1-1 Minami-ohsawa, Hachiohji-shi, Tokyo 192-0397, Japan

takagi-shinsuke@tmu.ac.jp

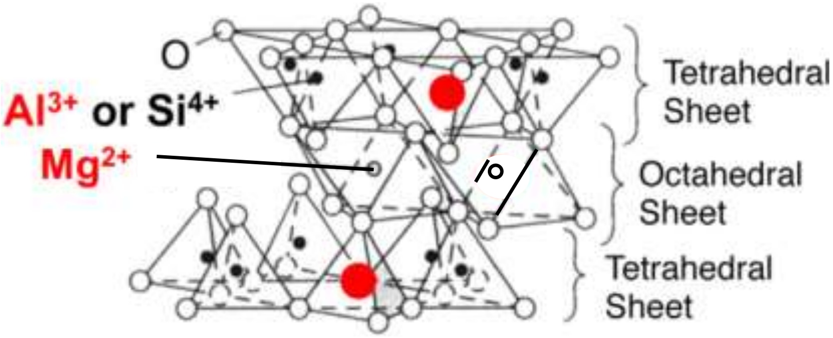

Figure S1. The unit structure of saponite.

Table S1. Chemical Formulas of Synthetic Saponites.

Synthetic saponite	$[(Si_{8-x}Al_x)(Mg_{6-y}Al_y)O_{20}(OH)_4]^{-(x-y)}$		
	x	y	$x-y$
Sap1.0	1.03	0	1.03
Sap1.2 (SSA)	0.80	0.03	0.77
Sap1.4	0.56	0.03	0.53
Sap1.6	0.45	0	0.45

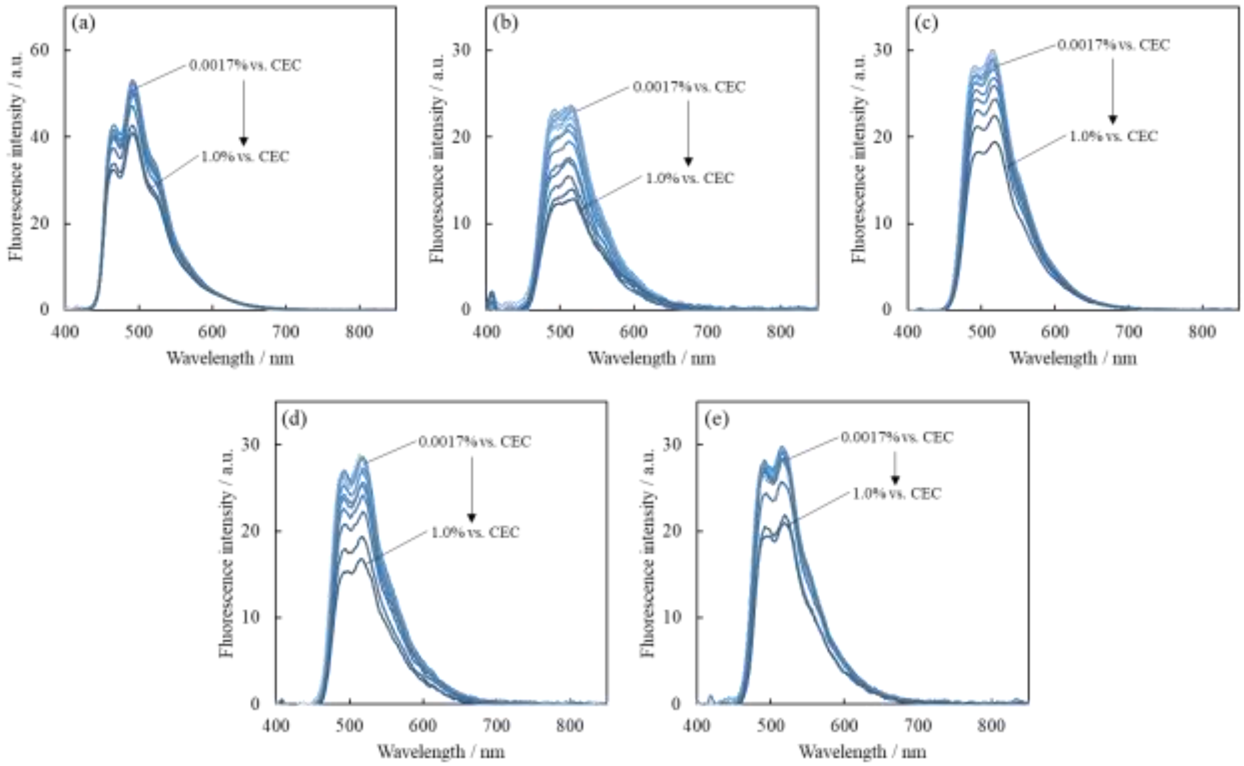

Figure S2. Fluorescence spectra of Acr-clay complexes: (a) Acr^+/Sap1.2, (b) $PhAcr^+$/Sap1.0, (c) $PhAcr^+$/Sap1.2, (d) $PhAcr^+$/Sap1.4 and (e) $PhAcr^+$/Sap1.6 in water. [Acr] = 3.33×10^{-9} M, [Sap] = 2.0×10^{-4}–3.33×10^{-7} equiv. L^{-1}. The dye loading levels were 0.0017, 0.0024, 0.0032, 0.0048, 0.0075, 0.010, 0.017, 0.025, 0.033, 0.050, 0.075, 0.10, 0.25, 0.50 and 1.0% vs. CEC of the clay. The excitation wavelength was 360 nm.

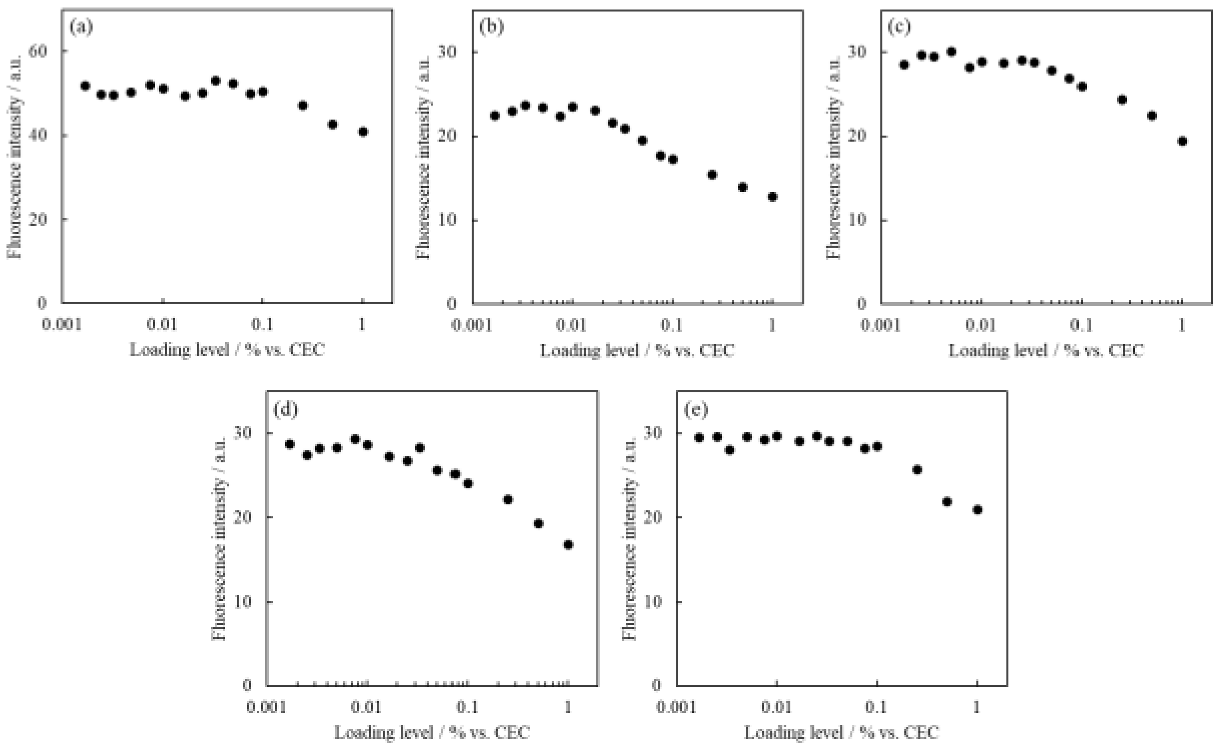

Figure S3. Fluorescence intensity-dye loading level plots of Acr-clay complexes: (a) Acr^+/Sap1.2, (b) $PhAcr^+$/Sap1.0, (c) $PhAcr^+$/Sap1.2, (d) $PhAcr^+$/Sap1.4 and (e) $PhAcr^+$/Sap1.6 in water. [Acr] = 3.33×10^{-9} M, [Sap] = 2.0×10^{-4}–3.33×10^{-7} equiv. L^{-1}. The dye loading level were changed from 0.0017 to 1.0% vs. CEC of the clay.

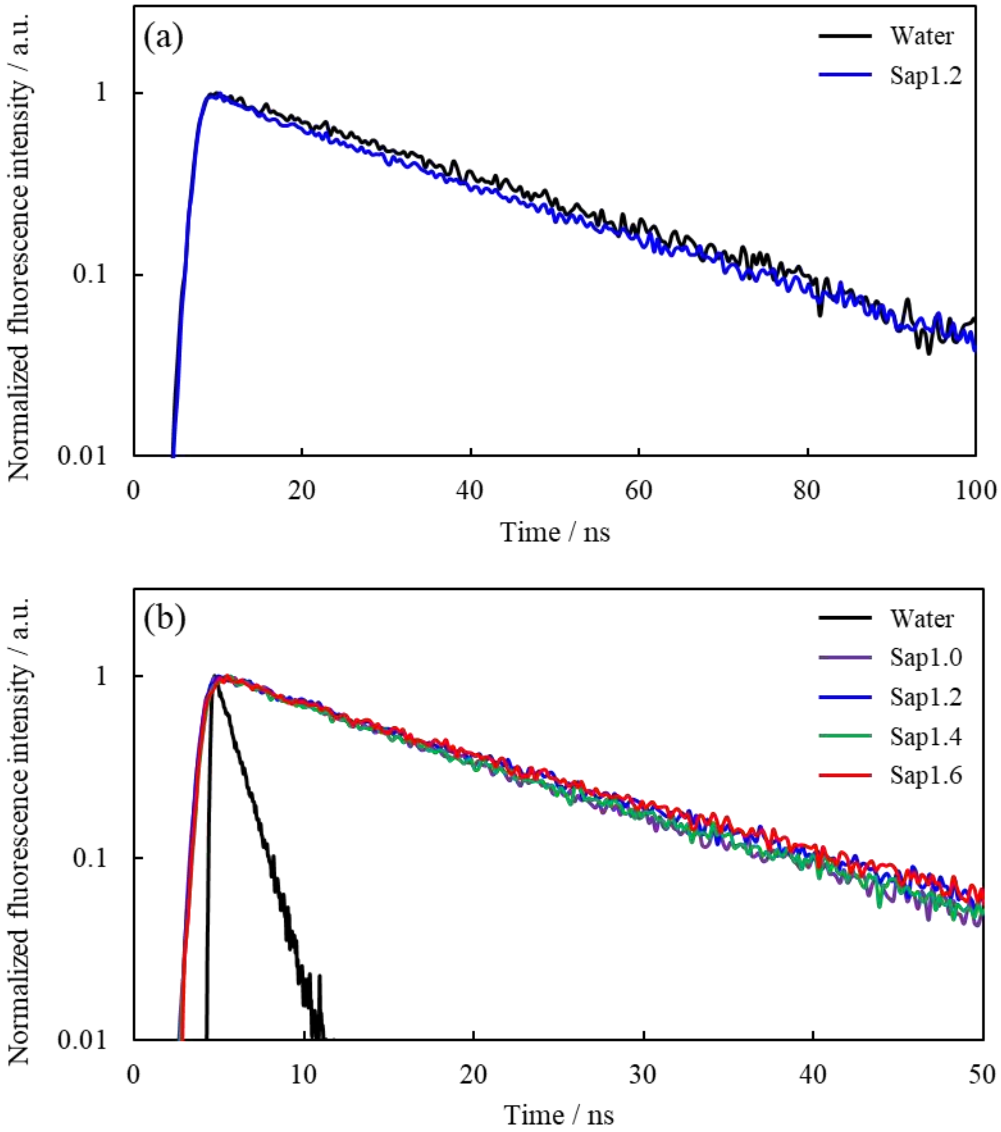

Figure S4. Fluorescence decay profile for (a) Acr^+ and (b) $PhAcr^+$ with and without various clays. [Acr] = 4.0×10^{-8} M, [Sap] = 4×10^{-4} equiv. L^{-1}. The dye loadings on the clay minerals were 0.01% vs. CEC.
